# Starter culture-related changes in free amino acids, biogenic amines profile, and antioxidant properties of fermented red beetroot grown in Poland

**DOI:** 10.1038/s41598-022-24690-9

**Published:** 2022-11-21

**Authors:** Renata Choińska, Katarzyna Piasecka-Jóźwiak, Łukasz Woźniak, Olga Świder, Elżbieta Bartosiak, Marzena Bujak, Marek Łukasz Roszko

**Affiliations:** 1 Department of Fermentation Technology, Prof. W. Dąbrowski Institute of Agricultural and Food Biotechnology-State Research Institute, Rakowiecka Str. 36, 02-532 Warsaw, Poland; 2 Department of Fruit and Vegetable Product Technology, Prof. W. Dąbrowski Institute of Agricultural and Food Biotechnology-State Research Institute, Rakowiecka Str. 36, 02-532 Warsaw, Poland; 3 Department of Food Safety and Chemical Analysis, Prof. W. Dąbrowski Institute of Agricultural and Food Biotechnology-State Research Institute, Rakowiecka Str. 36, 02-532 Warsaw, Poland

**Keywords:** Microbiology, Health care

## Abstract

Fermentation of two red beet cultivars (Wodan and Alto) with single-strain starter cultures consisting of selected strains of lactic acid bacteria (LAB) of plant origin (*Weissella cibaria* KKP2058, *Levilactobacillus brevis* ZF165) and a multi-strain culture (containing *W. cibaria* KKP2058, *L. brevis* ZF165, *Lactiplantibacillus plantarum* KKP1822, *Limosilactobacillus fermentum* KKP1820, and *Leuconostoc mesenteroides* JEIIF) was performed to evaluate their impact on betalains, free amino acids, formation of biogenic amines, and antioxidative properties of the juice formed. Next-generation sequencing data analysis used to identify the microbial community revealed that the starter cultures promoted the dominance of the genus *Lactobacillus*, and decreased the proportion of spoilage bacteria compared to spontaneously fermented juices. Generally, the fermentation process significantly influenced the amount of the analyzed compounds, leading in most cases to their reduction. The observed changes in the studied parameters depended on the starter culture used, indicating different metabolic activities of the LAB strains towards bioactive compounds. The use of multi-strain starter cultures allowed to largely prevent the reduction of betacyanins and histamine formation.

## Introduction

Red beetroot (*Beta vulgaris* sp.) is a popular vegetable having high nutritional value owing to its beneficial composition. It is a source of valuable bioactive compounds such as betalains, polyphenols, dietary fiber, and vitamins. Many reports describe the beneficial impact of betalains or polyphenols due to antioxidant activity in the prevention of chronic non-infectious diseases, the etiology of which is directly related to oxidative stress^1^. Their nutritional effectiveness is determined by the bioaccessibility and bioavailability that are in turn affected by several factors including composition and physicochemical properties of the digested food matrix, pH, temperature, and stability of the compounds during food processing^2^. Red beetroot can be consumed in its fresh form but mostly undergoes either thermal treatments *e.g*. boiling or dehydration process *i.e.* lyophilization (as food additives) which alters the original content of nutrients. Another common approach in red beet processing is lactic fermentation, which is a natural process involving lactic acid bacteria (LAB) to preserve fruits and vegetables. The preservative effect of LAB is a complex phenomenon consisting of various LAB activities, *i.e.* the production of organic acids such as lactic acid, acetic acid, and propionic acid causing acidification of the environment, and other antimicrobial metabolites such as hydrogen peroxide, bacteriocins, and low molecular weight metabolites. The metabolic activity of LAB leading to modification of the microenvironment enables them to compete with other co-inhabiting microorganisms and decrease their population. This is especially important in the case of harmful microorganisms responsible for food spoilage and the production of undesirable compounds, *e.g*. mycotoxins, biogenic amines (BA). However, it has been observed that the presence of native LAB is not always sufficient to ensure the proper control of the fermentation process due to *e.g*. the poor quality of raw materials, a limited number of LAB, inadequate processing and storage conditions, and therefore, do not guarantee the same efficiency of the fermentation process, and ensuring the desired quality and microbiological safety of the final product. The use of selected strains of LAB in form of starter cultures is therefore preferable because they effectively take control over the process, improving the nutritional quality and shelf-life of the product^3,4^. Starter cultures not only modify the physicochemical composition of the fermented materials, providing products with more diverse sensory and health properties but also affect the microbial community^5–8^. In a study by Jung et al.^9^, *Leuconostoc mesenteroides* used as a starter for kimchi fermentation led to an increase in the proportion of *Leuconostoc* and a decrease in the proportion of *Lactobacillus* as the starter fermented faster and produced more organic acid and other metabolites. The effect of fermentation on bioactive compounds like betalains or polyphenols, as well as on the antioxidant activity of red beet products has been a subject of many previous studies, in which it was reported that fermentation effectively affects the amounts of these compounds^10–12^. Recently, the use of LAB starter culture to control the accumulation of BA in fermented food becomes more important^13^. BA are nitrogen compounds, naturally occurring in microorganisms, plants, and animals, with high biological activity and that can exert a harmful effect on human health when consumed in excess. They are formed through the decarboxylation of their respective amino acid precursors by various microorganisms through substrate-specific decarboxylase enzymes or by amination and transamination of aldehydes and ketones^14,15^. For example, tyramine, histamine, phenylethylamine, tryptamine, and cadaverine are formed from tyrosine, histidine, phenylalanine, tryptophan, and lysine, respectively^16^. BA are nitrogen sources and precursors for synthesizing hormones, alkaloids, nucleic acids, and proteins. Moreover, they participate in many physiological processes e.g. protection against oxidative stress, biofilm formation, signaling, virulence, response to biotic and abiotic stress, and in the case of fermentation in acid stress response mechanism. BA can be found both in raw and fermented foods such as meat, dairy products, fish, wine, and vegetables^17^. It has been shown that fermented products, especially those obtained during spontaneous fermentation, have a higher BA content than raw food, resulting from the metabolic activity of the native microflora. The capability to form BA has been observed in both gram-positive bacteria *e.g*. *Lactobacillus*, *Lactococcus*, *Enterococcus,* and gram-negative species of the families *Enterobacteriaceae and Pseudomonadaceae*^18,19^. The presence of spoilage bacteria in the spontaneously fermented product proving that the fermentation failed, contributes to the overall increase of the BA. Marino et al.^20^ found a positive correlation between the count of studied *Enterobacteriaceae* species and the concentration of selected BA in the product.

In addition to determining the content of biogenic amines, it is also important to analyze free amino acid content. Free amino acids are not only precursors of many bioactive compounds such as protein or BA, but they impair also the taste, nutritional value, and antioxidant properties of the fermented products^18^. A recent study on the free amino acids profile of fermented cucumber, kimchi, and fermented soybean foods revealed its changes depending on the LAB strain used^21–23^. Literature data on the content of free amino acids in fermented red beets are scarce.

Given the growing consumer interest in fermented red beet products with desirable sensory properties and microbiologically safe, further research is needed to assess the role of fermentation microbiota on produced metabolites when designing starter cultures.

In this context, this work aimed to compare the effect of selected LAB starter cultures on betalains, amino acid composition, biogenic amine formation, and antioxidant potential of juices obtained during red beet fermentation to assess the starter cultures—related changes. For this purpose, fermentation of two red beet cultivars (Wodan and Alto) grown in Poland with selected LAB strains of plant origin in form of single and mixed starter cultures was performed. Next-generation sequencing data analysis was used to analyze the diversity of the microbial community due to the fermentation consortia formed after using a starter culture.

## Results and discussion

### pH

**Figure 1:**
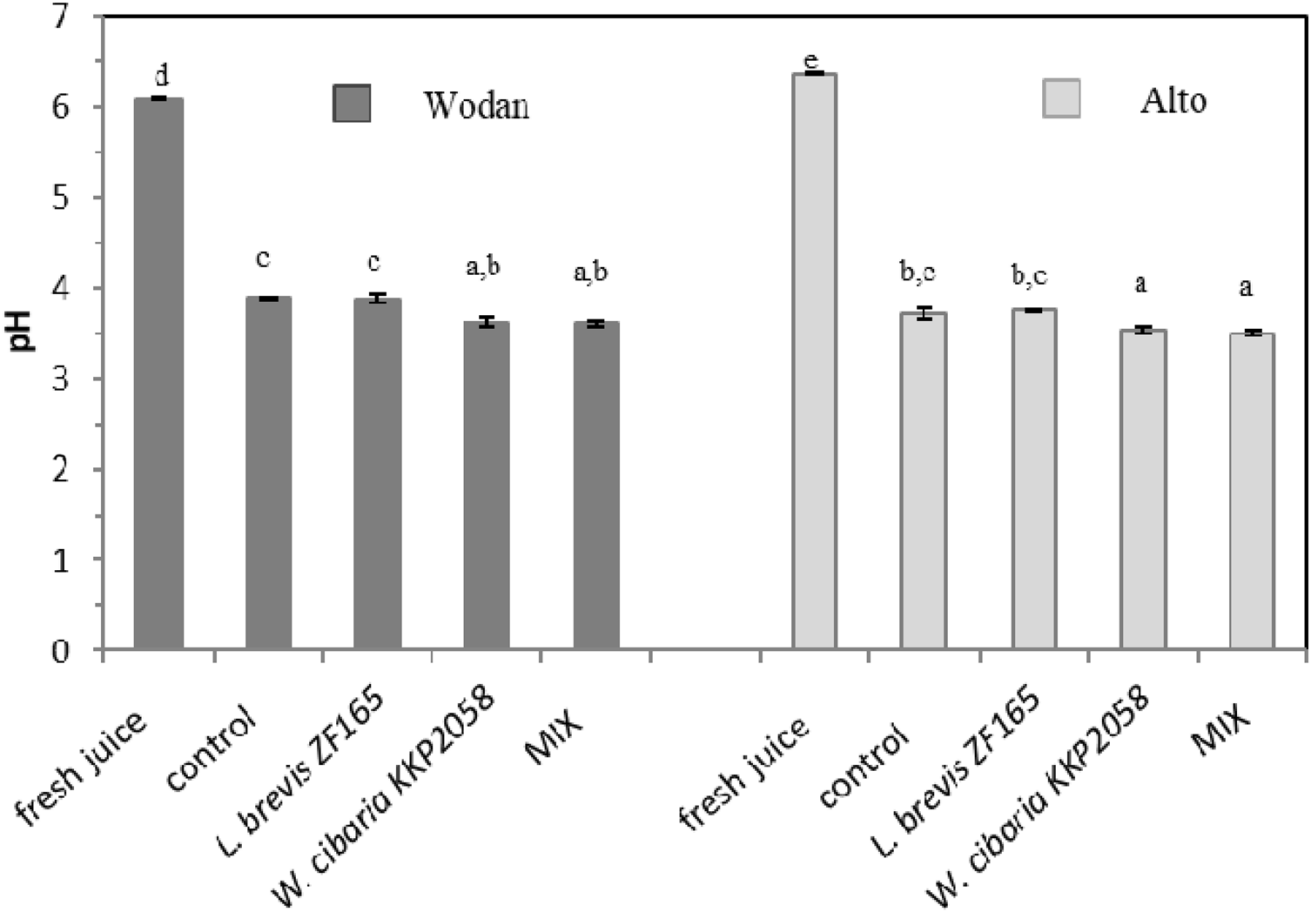
pH of fresh and fermented juices of Wodan and Alto variety. MIX *i.e.* five-strain starter culture (containing *W. cibaria* KKP2058, *L. brevis* ZF165, *L. plantarum* KKP1822, *L. fermentum* KKP1820, and *Leuconostoc mesenteroides* JEIIF). The results are expressed as mean ± SD. Different letters show significant differences between obtained values (*p* < 0.05), according to Tukey’s HSD test. Control refers to samples fermented spontaneously with native microflora.

Two red beet cultivars *i.e.* Alto and Wodan, were fermented with single-strain starter culture consisting of selected LAB strains of plant origin *i.e. W. cibaria* KKP2058, and *L. brevis* ZF165, and a mixed-strain culture (MIX) containing *W. cibaria* KKP2058, *L. brevis* ZF165, *L. plantarum* KKP1822, *L. fermentum* KKP1820, and *Leuconostoc mesenteroides* JEIIF. Uninoculated red beetroots (spontaneous fermentation) were prepared parallel and served as a control. After 7 days of fermentation, the initial pH of 6.08 (Wodan) and 6.36 (Alto) considerably dropped and was in the range of 3.61–3.89 and 3.50–3.75, for Wodan and Alto respectively (Fig. 1). The lowest pH was obtained in the juices fermented with a single-strain culture of *W. cibaria* KKP2058 and MIX, independently of the cultivar.

### Microbial community composition of the fermented red beetroots

**Figure 2:**
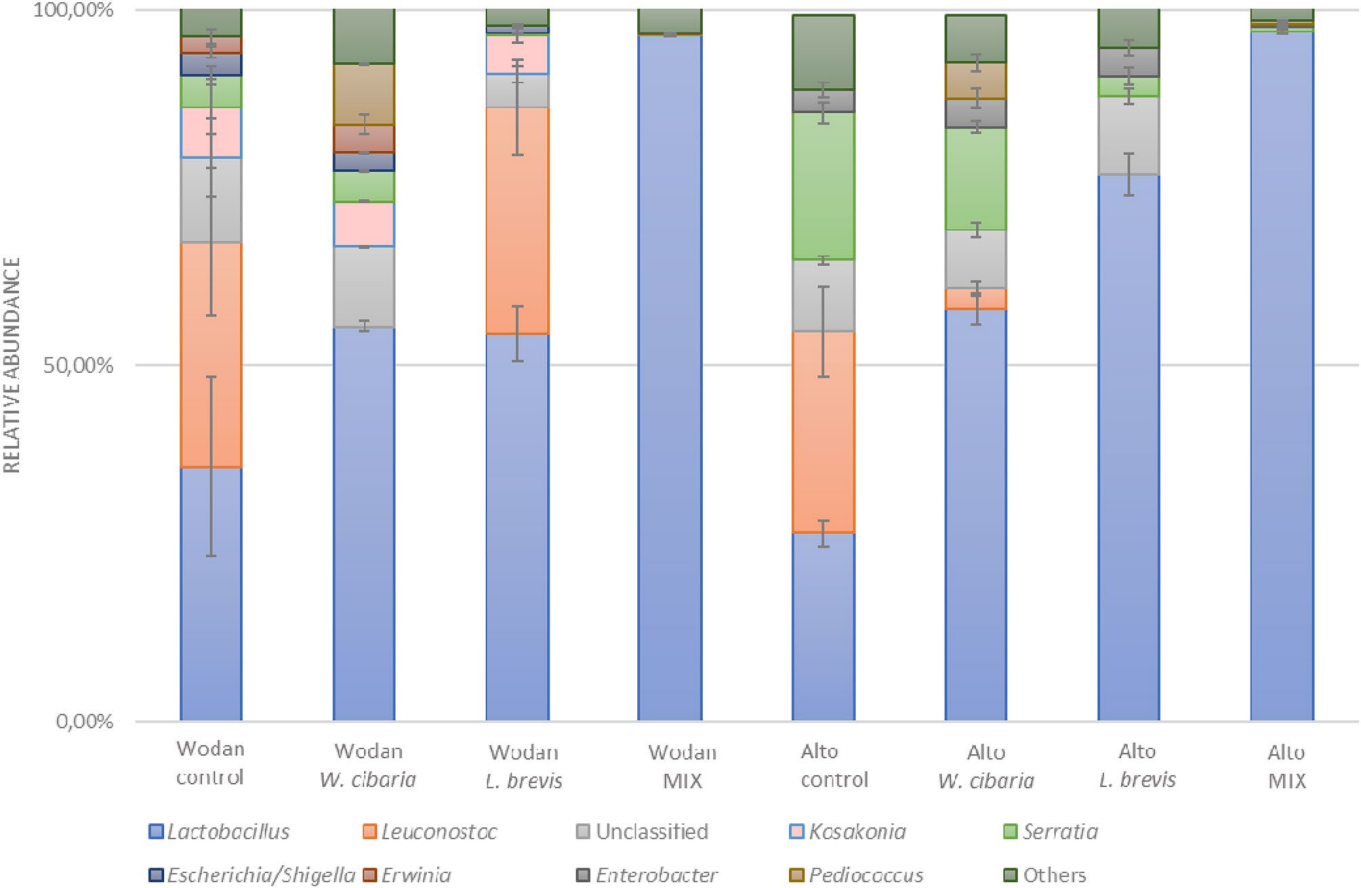
The microbial community of fermented juices of Wodan and Alto variety at the genus level revealed by high-throughput sequencing. Control denotes spontaneously fermented samples. A relative abundance of bacteria genera accounted for below 1% has not been shown. The name *Lactobacillus* refers to the old nomenclature, and therefore acc. to the current classification it includes 25 genera *i.a. Lactobacillus*, *Lacticaseibacillus*, *Lactiplantibacillus* (including *L. plantarum*), *Levilactobacillus* (including *L. brevis*), *Limosilactobacillus* (including *L. fermentum*)^25^.

Microbial community assessment is a key factor in understanding the role of microorganisms on the studied characteristics of fermented samples. The use of next-generation sequencing techniques is nowadays, a powerful approach allowing for exploring the composition and diversity of complex microbial communities^24^. Microbial community analysis of raw and fermented beetroot juices showed differences both in terms of genera and their relative abundance between samples (Fig. 2). The composition of the raw juices consisted mainly of *Streptophyta* species (70–74%). *Lactobacillus* accounted for approximately 0.2% (data not shown).

The relative abundance of the genus formerly known as *Lactobacillus* in samples treated with starter cultures accounted for more than 50% of the total bacteria and was higher than that of spontaneous fermentation. The highest relative abundance *i.e.* 97% of *Lactobacillus* was detected in juices obtained during fermentation with a mixed-strain culture independently of the cultivar used. This finding suggests that the use of the multi-component starter culture allowed lactobacilli to dominate at the end of fermentation and limit the potential plant pathogens. The juices treated with single-strain starter cultures showed more diverse bacterial composition including *Lactobacillus* (55–76%), *Leuconostoc* (3–32%), *Pediococcus* (6–8%), *Kosakonia* (5–6%)*, Serratia* (4–14%), and *Enterobacter* (4%). The observed differences in the proportion of detected bacteria genera and the presence of plant pathogens, members of *Enterobacterales* family would suggest that the single-strain starter cultures did not fully control the micro-environment. Moreover, in the case of samples inoculated with *W. cibaria* KKP2058 the relative abundance of the *Weissella* genus was lower than 1%. A minimal relative abundance of *Weissela* spp. in fermented with starter culture Chinese Sichuan sausages at the end of fermentation was observed also by Ren et al.^13^. The microbial community of spontaneously fermented beetroots was dominated by two microbial groups: *Lactobacillus* (36%), followed by *Leuconostoc* (32%) for the Wodan cultivar, and *Leuconostoc* (28%), followed by *Lactobacillus* (26%) for Alto cultivar. In these cases, species belonging to *Kosakonia*, *Serratia*, and *Enterobacter* were also identified but their relative abundance varied between the cultivars. Alto was characterized by a higher content of gram-negative bacteria belonging to *Serratia* (21% vs. 3%), while the microbial community of Wodan consists of other gram-negative bacteria, *Kosakonia*, that was not identified in the Alto cultivar.

The analysis of the relative abundance of lactobacilli at the species level (results not shown) revealed that the use of *L. brevis* ZF165 as a starter increased the *L. brevis* proportion in both fermented cultivars. Similarly, in the samples inoculated with MIX this species was most abundant followed by *Lactobacillus* sp. and *L. fermentum*.

Thus, our results confirm the previous findings indicating that the addition of starter culture can effectively affect the microbial community diversity during fermentation due to the interspecific competition. Compared with spontaneous fermentation, inoculation led to an increase in the relative abundance of lactobacilli and a decrease in the relative abundance of pathogens in single-strain cultures. In the case of mixed cultures, the pathogens were barely detectable.

### Betalains

Generally, three red-violet colorants (betacyanins) *i.e.* betanin, isobetanin, neobetanin, and two yellow colorants (betaxanthins) *i.e.* vulgaxanthins I, vulgaxanthins II, were identified in the analyzed fresh juices of Wodan and Alto cultivars. The obtained MS data are presented in Table S1. The total concentration of betalains found in fresh juices of Wodan was around 890 mg·L^−1^, and 937 mg·L^−1^ was obtained from the Alto variety (Table 1).

**Table 1:** Total content and quantitative composition of betalains found in fresh and fermented red beet juices of Wodan and Alto variety.

Sample	Betaxanthins		Betacyanins						Total amount of betaxanthins [mg·L^−1^]	Total amount of betacyanins [mg·L^−1^]
	Vulgaxanthin I [mg·L^−1^]	Vulgaxanthin II [mg·L^−1^]	2-O’-glucosyl-betanin [mg·L^−1^]	Betanin [mg·L^−1^]	Isobetanin [mg·L^−1^]	Betanidin [mg·L^−1^]	Isobetanidin [mg·L^−1^]	Neobetanin [mg·L^−1^]		
Wodan (fresh)	99.7±7.4^b^	5.57±0.77^b^	nd	670.10±6.81^d^	67.80±1.62^c^	nd	nd	46.80±1.30^e^	105.27	784.7
Wodan (control)*	17.53±0.17^a^	1.37±0.27^a^	2.53±0.32^a,b^	46.00±1.91^a^	2.50±1.01^a^	202.93±20.14^d^	11.80±0.91^b^	1.83±0.32^a,b^	18.90	267.59
Wodan (*L. brevis* ZF165)	15.20±2.05^a^	11.70±0.14^d^	4.53±1.56^b,c^	38.57±13.82^a^	7.57±2.70^a^	184.27±53.17^c,d^	11.47±3.44^b^	3.07±0.84^b,c^	26.90	249.48
Wodan (*W. cibaria* KKP2058)	11.23±0.52^a^	11.40±0.36^d^	10.0±0.20^d^	141.67±17.47^b^	15.20±1.33^a^	71.30±8.68^a,b^	5.10±1.15^a,b^	5.27±0.18^c^	22.63	248.54
Wodan (MIX)**	9.13±0.18^a^	7.30±0.03^b,c^	7.43±1.69^c,d^	300.77±4.91^c^	38.33±2.02^b^	18.53±1.51^a^	1.33±0.14^a^	8.27±0.50^d^	16.43	374.66
Alto (fresh)	118.17^±4.79c^	9.03^±1.12c,d^	nd	675.07^±12.01d^	78.83^±8.67c^	nd	nd	55.70^±1.00f.^	127.2	809.60
Alto (control)*	5.40±0.95^a^	0.47±0.18^a^	2.07±0.50^a,b^	39.20±0.64^a^	3.57±1.69^a^	107.35±2.55^b,c^	8.65±0.15^a,b^	0.17±0.09^a^	5.87	161.01
Alto (*L. brevis* ZF165)	11.43±0.35^a^	11.10±0.11^d^	1.13±0.17^a,b^	31.53±1.58^a^	4.10±0.21^a^	163.97±14.12^b,c,d^	9.13±1.12^b^	0.27±0.09^a^	22.53	210.13
Alto (*W. cibaria* KKP2058)	7.07±1.13^a^	7.90±0.50^b,c^	7.87±0.54^c,d^	120.30±9.55^b^	14.97±0.94^a^	89.13±2.57^a,b,c^	6.20±1.04^a,b^	0.80±0.17^a,b^	14.97	239.27
Alto(MIX)**	7.63±0.32^a^	7.93±0.03^b,c^	3.30±0.47^a,b^	284.47±8.20^c^	43.43±6.79^b^	11.40±0.47^a^	1.20±0.20^a^	1.13±0.18^a,b^	15.56	344.93

*Control: spontaneous fermentation.

**MIX: *L. brevis* ZF165; *W. cibaria* KKP2058; *L. plantarum* KKP1822; *L. fermentum* KKP1820; *Leuconostoc mesenteroides* JEIIF.

Different letters indicate significant differences among the mean values according to the least significant difference test (*P* ≤ 0.05).

The obtained concentrations differ from those reported for fresh juices of other varieties cultivated in Poland (1270 mg·L^−1^ and 854 mg·L^−1^ for Chrobry and Czerwona Kula, respectively) indicating the diversity of the red beetroots regarding the concentration of betalains^26^. The dominant red-violet colorant was betanin, whose content was estimated to be approximately 85% for Wodan and 83% for Alto of the total betacyanins. Among the yellow colorants prevailed vulgaxanthin I (95% and 93%, for Wodan and Alto, respectively) (Table 1). The cultivar Alto was characterized by a slightly higher content of vulgaxanthis I (118.2 mg·L^−1^) than Wodan (99.7 mg·L^−1^). The composition of betalains changed remarkably after fermentation and was mainly strain-dependent. A drastic decrease in the content of both red and yellow colorants and the formation of new compounds such as 2-O’-glucosylbetanin and aglycons of betanin and isobetanin i.e. betanidin and isobetanidin was observed. The formation of these compounds during LAB fermentation was previously reported by other authors^11,26^. According to the literature, the conversion of betanin and its isomer to the corresponding aglycons occurs by their hydrolysis catalyzed by the bacterial enzyme ß-glucosidase. Spontaneous fermentation and that performed with *L. brevis* ZF165 caused the highest degradation of betanins and the highest increase in the content of their aglycons, regardless of the cultivar used. The samples fermented with MIX were characterized by the highest level of betanin (300.77 and 284.47 mg·L^−1^ for Wodan and Alto, respectively), isobetanin (38.33 and 43.43 mg·L^−1^ for Wodan and Alto, respectively), and neobetanin (8.27 and 1.13 mg·L^−1^ for Wodan and Alto, respectively) and the lowest level of the aglycons: betanidin (18.53 and 11.40 mg·L^−1^ for Wodan and Alto, respectively) and isobetanidin (1.33 and 1.20 mg·L^−1^ for Wodan and Alto, respectively). Among the single-strain starters, an interesting behavior in the content of betanin and isobetanin and their aglycons was observed. The juices obtained during fermentation with *W. cibaria* KKP 2058 had a much higher level of betanin (141.67 mg·L^−1^ vs. 38.57 mg·L^−1^ and 120.30 mg·L^−1^ vs. 31.53 mg·L^−1^ for Wodan and Alto, respectively) and isobetanin (15.20 mg·L^−1^ vs. 7.57 mg·L^−1^ and 14.97 mg·L^−1^ vs. 4.10 mg·L^−1^ for Wodan and Alto, respectively) than those with *L. brevis* ZF165. Whereas, *L. brevis* ZF165 characterized higher content of betanidin (184.27 mg·L^−1^ vs. 71.30 mg·L^−1^ and 163.97 mg·L^−1^ vs. 89.13 mg·L^−1^ for Wodan and Alto, respectively) and isobetanidin (11.47 mg·L^−1^ vs. 5.10 mg·L^−1^ and 9.13 mg·L^−1^ vs. 6.20 mg·L^−1^ for Wodan and Alto, respectively). In the case of neobetanin and 2-O’glucosylbetanin, the juices obtained during fermentation with *W. cibaria* KKP 2058 showed a higher level of these colorants than those inoculated with *L. brevis* ZF165. In addition, the level of neobetanin in juices of all fermented samples of the Wodan cultivar was in the range from 1.83 mg·L^−1^ up to 8.27 mg·L^−1^ and higher than in fermented Alto juices (0.17–1.13 mg·L^−1^). An opposite relation between both cultivars was observed in the raw juices (46.80 mg·L^−1^ vs. 55.70 mg·L^−1^ for Wodan and Alto, respectively). In terms of betaxanthin levels, the largest decrease, of around 90%, was observed for the vulgaxanthin I regardless of the cultivar. A similar finding was reported by Sawicki & Wiczkowski^11^, who compared the effect of boiling and spontaneous fermentation on the profile and content of betalains of red beetroot of the cultivar Czerwona Kula. The lowest amount of vulgaxanthin I was found in juices of Alto fermented with *W. cibaria* KKP 2058 (7.07 mg·L^−1^) and MIX (7.63 mg·L^−1^), and for Wodan, in sample fermented with MIX (9.13 mg·L^−1^). In the case of vulgaxanthin II, its greatest decrease was observed in spontaneously fermented samples. The fermented samples were less affected and even the Wodan and Alto samples fermented with *L. brevis* ZF165 showed a twofold increase in the concentrations of vulgaxanthin II. The obtained results clearly show the diversity among the used starter cultures in terms of their ability to the transformation of the main red beet colorants. Generally, single starter cultures had a higher betanin hydrolysis ability than the multi-culture starter, and *L. brevis* ZF165 showed the highest betanin conversion resulting mainly from endogenous β-glucosidase activity. This finding has been confirmed by the calculated acc. to Czyżowska et al.^26^ β-glucosidase index (Table S2), which was the lowest among fermented juices indicating higher activity of β-glucosidase of *L. brevis* ZF165 compared to the other LAB strains. The calculated isobetanin/betanin ratio (I/B), related to the isomerization activity, was the lowest in the case of spontaneously fermented juices (0.05 and 0.09 for Wodan and Alto, respectively). The fermented juices characterized almost similar values of I/B, except *L. brevis* ZF165 Wodan, having the highest I/B (0.20). The neobetanin/betanin ratio, N/B, showing the dehydrogenase activity, was the highest in the fresh juices and that of Wodan inoculated with *L. brevis* ZF165. The highest vulgaxanthin I/betanin ratio (V/B) was obtained for Wodan and Alto inoculated with *L. brevis* ZF165 and spontaneously fermented Wodan. The fermented with *W. cibaria* KKP2058 and MIX samples characterized the lowest V/B regardless of the cultivar used, indicating better stability of betanin during fermentation.

Table 2 summarizes the calculated index for *L. brevis* strains used for fermentation of various red beet cultivars grown in Poland. The observed different activities in betalains conversion were cultivar and used strain-dependent.

**Table 2:** Comparison of betalains changes after fermentation expressed by betanin + isobetanin/betanidin + isobetanidin, B + I/Bd + Id, isobetanin/betanin, I/B, neobetanin/betanin, N/B, and vulgaxanthin I/betanin, V/B, ratios for fermented red beetroots of varied cultivars grown in Poland.

Strain	Cultivar	B + I/Bd + Id	I/B	N/B	V/B
*L. brevis* ZF165	Wodan	0.23	0.20	0.08	0.39
*L. brevis* ZF165	Alto	0.20	0.13	0.008	0.36
*L. brevis* 0944^26^	Chrobry	0.34	0.28	0.14	0.81
*L. brevis* 0944^26^	Czerwona Kula	3.14	0.23	0.11	0.16

### Free amino acids

A total number of 17 free amino acids were identified in the studied fresh red beet juices of the Wodan and Alto cultivar (Table 3).

**Table 3:** Content of free amino acids in the fresh and fermented red beet juices of Wodan and Alto variety.

Amnio acid [mg·L^−1^]	Wodan (fresh)	Wodan (control)*	Wodan (*L. brevis* ZF165)	Wodan (*W. cibaria* KKP2058)	Wodan (MIX)**	Alto (fresh)	Alto (control)*	Alto (*L. brevis* ZF165)	Alto (*W. cibaria* KKP2058)	Alto(MIX)**
Glutamine	5878.06 ± 177.50f.	4119.30 ± 191.23^e^	3713.36 ± 157.49^d,e^	3495.07 ± 108.27^c,d^	3127.15 ± 64.16^b,c^	7960.52 ± 145.91^ g^	2381.67 ± 46.96^a^	2473.39 ± 34.23^a^	2883.79 ± 117.10^a,b^	2650.69 ± 27.19^a,b^
Isoleucine	267.62 ± 6.41^e^	202.86 ± 14.86^d^	173.47 ± 2.49^a^	154.46 ± 2.39^a^	151.93 ± 1.59^a,c^	304.44 ± 6.15f.	100.43 ± 0.91^b^	156.32 ± 2.35^a^	128.71 ± 1.98^c^	91.28 ± 0.78^b^
Leucine	164.41 ± 4.05^d^	135.07 ± 2.70^b^	127.93 ± 1.38^a,b^	156.55 ± 3.51^d^	136.03 ± 1.50^b^	210.84 ± 2.87^e^	96.80 ± 1.20^c^	123.44 ± 1.52^a^	119.66 ± 2.30^a^	98.72 ± 0.71^c^
Ornithine	1.50 ± 0.13^a^	0.90 ± 0.18^a^	77.33 ± 8.25^b^	75.05 ± 5.81^b^	83.66 ± 4.35^b^	0.89 ± 0.07^a^	7.80 ± 0.85^a^	191.76 ± 5.09^d^	245.63 ± 13.16^c^	245.37 ± 11.06^c^
Valine	139.19 ± 3.64^d,f^	130.08 ± 12.94^d^	114.55 ± 1.79^b,d^	103.04 ± 2.31^a,b^	95.82 ± 1.88^a,b,c^	169.86 ± 2.02f.	80.84 ± 1.58^c,e^	105.27 ± 1.48^a,b^	84.49 ± 2.46^a,c^	59.47 ± 0.39^e^
Threonine	73.54 ± 2.23^ g^	56.76 ± 2.83f.	49.21 ± 0.70^d^	40.26 ± 0.50^a,b,c^	41.41 ± 0.59^b,c^	112.37 ± 1.93^ h^	35.37 ± 0.66^a^	45.83 ± 0.59^c,d^	27.12 ± 1.34^e^	36.74 ± 0.25^a,b^
Arginine	142.38 ± 4.51^b^	1.22 ± 0.06^a^	nd	1.97 ± 0.10^a^	1.76 ± 0.18^a^	842.76 ± 16.86^d^	26.67 ± 2.76^c^	nd	2.63 ± 0.13^a^	1.79 ± 0.04^a^
Tyrosine	15.06 ± 0.58^b^	6.97 ± 0.55^b^	1.57 ± 0.11^a^	6.79 ± 0.23^a,b^	3.35 ± 0.27^a^	36.29 ± 0.48^c^	2.64 ± 0.24^a^	0.99 ± 0.08^a^	1.54 ± 0.01^a^	2.82 ± 0.12^a^
Lysine	23.63 ± 0.24^d,e^	11.18 ± 0.13^c^	13.00 ± 0.39^a,c^	34.85 ± 1.53f.	28.29 ± 1.12^e^	15.93 ± 0.32^a,b^	5.55 ± 0.82f.	16.57 ± 1.17^a,b^	20.26 ± 0.17^b,d^	16.86 ± 0.29^a,b^
Phenylalanine	14.43 ± 0.52^b^	30.97 ± 0.86f.	12.00 ± 2.09^b^	6.69 ± 0.68^c^	7.66 ± 0.33^c,d^	20.61 ± 0.42^e^	nd	11.15 ± 0.66^b,d^	nd	nd
Histidine	21.61 ± 3.50^d,e^	37.95 ± 1.38^b^	33.31 ± 2.49^a,b^	31.05 ± 1.37	25.76 ± 0.42^c,d,e^	38.52 ± 5.70^a,b^	32.36 ± 1.92^a,b,c^	33.31 ± 0.87^a,b,c^	28.83 ± 1.47^a,b,c^	22.60 ± 0.65^d^
Asparagine	489.02 ± 13,57^e^	259.06 ± 8.97^a^	257.12 ± 2.17^a^	292.04 ± 9.62^a,b^	270.2 ± 3.59^a,b^	797.17 ± 13.25f.	91.08 ± 16.80^c^	209.38 ± 2.06^d^	282.05 ± 10.16^a,b^	301.08 ± 1.46^b^
Serine	333.91 ± 8.82^d^	240.55 ± 15.74^c^	175.65 ± 23.83^a^	134.83 ± 3.06^a^	4.35 ± 0.84^b^	407.89 ± 7.29^e^	176.75 ± 2.68^a^	175.90 ± 1.93^a^	172.15 ± 2.72^a^	15.79 ± 0.37^b^
Methionine	9.03 ± 0.14^b,c^	6.54 ± 0.70^a^	5.37 ± 0.22^a,g^	9.44 ± 0.24^c^	9.01 ± 0.18^b,c^	7.15 ± 0.07^a,b^	nd	1.69 ± 0.76^d,e^	4.30 ± 0.18f.^,g^	3.33 ± 0.03^e,f^
Proline	15.90 ± 0.20^d^	9.66 ± 0.43^a^	9.04 ± 0.08^a^	12.56 ± 0.17^b^	9.73 ± 0.13^a^	13.38 ± 0.13^b^	10.78 ± 0.08^c^	13.00 ± 0.30^b^	12.61 ± 0.12^b^	9.74 ± 0.07^a^
Aspartic acid	279.43 ± 7.11f.	73.55 ± 0.35^b^	136.22 ± 2.58^a^	104.04 ± 3.3^d^	92.86 ± 1.07^c,d^	422.20 ± 8.30^ g^	230.97 ± 4.64^e^	146.56 ± 1.70^a^	135.41 ± 4.32^a^	80.48 ± 0.85^b,c^
Glutamic acid	454.87 ± 14.71^d^	330.02 ± 20.66^a^	314.27 ± 2.28^a,c^	243.87 ± 10.32^b^	273.60 ± 8.72^a,b,c^	444.74 ± 11.52^d^	340.55 ± 18.79^a,e^	397.85 ± 26.32^d,e^	261.79 ± 7.66^b,c^	308.96 ± 2.17^a,b,c^
TAA	8323.60	5824.71	5149.20	4902.05	4374.60	11,805.57	3894.42	4111.07	4402.77	3934.70
EAA EAA/TAA	691.85 0.08	582.74 0.10	499.36 0.10	492.24 0.10	481.56 0.11	841.21 0.07	315.81 0.08	444.65 0.11	401.99 0.09	305.38 0.08
SAA SAA/TAA	349.81 0.042	250.21 0.043	184.69 0.036	147.39 0.030	14.08 0.003	421.27 0.036	187.53 0.048	188.90 0.046	184.76 0.042	25.53 0.006
FAA FAA/TAA	6367.08 0.76	4378.36 0.75	3970.48 0.77	3787.11 0.77	3397.35 0.78	8757.69 0.74	2472.75 0.63	2682.77 0.65	3165.84 0.72	2951.77 0.75
HAA HAA/TAA	610.58 0.07	517.29 0.09	440.47 0.09	442.59 0.09	421.59 0.10	726.29 0.06	288.86 0.07	410.89 0.10	348.56 0.08	261.56 0.07

*Control: spontaneous fermentation.

**MIX: *L. brevis* ZF165; *W. cibaria* KKP2058; *L. plantarum* KKP1822; *L. fermentum* KKP1820; *Leuconostoc mesenteroides* JEIIF. nd-not detected.

TAA-total amino acid; EAA- essential amino acids (sum of valine, methionine, phenylalanine, isoleucine, leucine, lysine, and threonine); SAA- sweet amino acid (sum of serine and proline); FAA- flavor amino acid (sum of glutamine and asparagine); HAA- hydrophobic amino acid (sum of proline, valine, methionine, phenylalanine, isoleucine, and leucine).

Different letters indicate significant differences among the mean values according to the least significant difference test (*P* ≤ 0.05).

Generally, the Alto cultivar was characterized by a higher value of total amino acids (TAA), essential amino acids (EAA), sweet amino acids (SAA), flavor amino acids (FAA), and hydrophobic amino acid (HAA) than Wodan. Glutamine dominated among the analyzed free amino acids and was 5878.06 mg·L^−1^, and 7960.52 mg·L^−1^ for Wodan and Alto, respectively. This is in line with previous observations showing that red beets are very high in glutamine^27^. The other amino acids with relatively high concentrations between 139.19 up to 489.02 mg·L^−1^ (Wodan) and 169.86 to 842.76 mg·L^−1^ (Alto) were valine, isoleucine, aspartic acid, leucine, serine, glutamic acid, and asparagine. Relatively lower concentrations 9.03–73.54 mg·L^−1^, for Wodan and 7.15–38.52 mg·L^−1^, for Alto were found for methionine, lysine, proline, phenylalanine, tyrosine, histidine, and threonine. The lowest concentrations were detected for ornithine (1.50 mg·L^−1^ and 0.89 mg·L^−1^, for Wodan and Alto, respectively). The fermentation process led in most cases to a significant reduction in the content of amino acids, except ornithine (Table 3). An enormous increase of ornithine from 0.89 mg·L^−1^ (in fresh juice) up to about 245 mg·L^−1^ was found especially for inoculated with *W. cibaria* and MIX red beets of Alto. In the case of Wodan, the ornithine level in the fermented with culture starters samples was between 75.05 and 83.66 mg·L^−1^. It is interesting to note, that the juices from both fermented cultivars were characterized by a simultaneously high drop in the content of arginine, from 142.38 and 842.76 mg·L^−1^ in fresh juices to 1.22 mg·L^−1^ and to 1.79 mg·L^−1^, for Wodan and Alto, respectively. Moreover, in the case of *L. brevis* ZF165 arginine was not detected at all in both cultivars. The observed large decrease in arginine content may be related to ornithine biosynthesis, in which arginine acts as a precursor, or to the formation of biogenic amine-putrescine. Arginine metabolism by wine and sourdough LAB through deaminase or arginase-urease pathway showed that only some heterofermentative lactobacilli e.g. *L. brevis*, were able to degrade arginine^28^. Another interesting observation made during this study concerned the content of lysine. Regardless of the variety, the juices fermented with *W. cibaria* KKP2058 and MIX red beets were characterized by a higher level of lysine compared to fresh juices. In contrast, the samples inoculated with *L. brevis* ZF165 and spontaneously fermented (control) showed a lower level of lysine. Thus, all observed changes in the content of amino acids were starter culture and cultivar-depended. Among the used inoculants, the greatest reduction in the amino acid content was caused by the preparations consisting of multi-culture strains (MIX).

### Biogenic amines

In the studied fresh red beet juices of Alto and Wodan cultivar a series of four biogenic amines (BA) *i.e.* spermine, spermidine, tyramine, and putrescine were detected (Table 4).

**Table 4:** Content of biogenic amines and the calculated BAI values for the fresh and fermented red beet juices of Wodan and Alto variety.

Sample	Spermine [mg·L^−1^]	Spermidine [mg·L^−1^]	Tyramine [mg·L^−1^]	Histamine [mg·L^−1^]	Cadaverine [mg·L^−1^]	Putrescine [mg·L^−1^]	Agmatine [mg·L^−1^]	2-phenylethylamine [mg·L^−1^]	Tryptamine [mg·L^−1^]	Total amount [mg·L^−1^]	BAI*** [mg·L^−1^]
Wodan (fresh)	0.27 ±0.04^a,b^	0.15±0.04^e^	0.63±0.02^a^	nd	nd	4.59 ±0.01^b,c,g^	nd	nd	nd	5.55	3.23
Wodan (control)*	0.17±0.01^a^	2.92±0.03^c,d^	4.36±0.08^b^	0.52±0.06^b^	1.02±0,33^c^	2.50±0.18^e,f^	nd	nd	nd	11.49	8.40
Wodan (*L. brevis* ZF165)	0.21 ±0.01^a,b^	2.98±0.03^c,d^	12.54 ±0.52^d^	0.25±0.03^a^	0.85±0.27^b,c^	3.41±0.46^b,f^	nd	nd	nd	20.24	17.05
Wodan (*W. cibaria* KKP2058)	0.16±0.01^a^	2.64±0.04^a,b^	4.38±0.07^b^	0.19±0.03^a^	0.17±0.01^a,b^	2.35±0.08^d,e^	nd	nd	nd	9.89	7.09
Wodan (MIX)**	0.21 ±0.03^a,b^	3.08±0.02^d^	8.68±0.45^c^	nd	0.15±0.02^a^	1.71±0.06^d^	nd	nd	nd	13.83	10.54
Alto (fresh)	0.32±0.07^b^	0.46±0.01^f^	0.61±0.01^a^	nd	nd	3.82 ±0.02^a,b,c^	nd	nd	nd	5.21	4.43
Alto (control)*	0.25 ±0.02^a,b^	2.65±0.06^a,b^	4.42±1.39^b^	0.21±0.01^a^	0.21±0.01^a,b^	4.50±0.31^c,g^	nd	nd	nd	12.24	9.34
Alto (*L. brevis* ZF165)	0.25 ±0.01^a,b^	2.19±0.08^g^	16.06 ±0.20^e^	0.41±0.03^b^	0.34 ±0.02^a,b,c^	5.48±0.08^g^	nd	nd	nd	24.73	22.29
Alto (*W. cibaria* KKP2058)	0.25 ±0.01^a,b^	2.48±0.04a	1.35±0.01^a^	nd	0.37 ±0.08^a,b,c^	3.62 ±0.14^a,b,c^	nd	nd	nd	8.07	5.34
Alto(MIX)**	0.26 ±0.01^a,b^	2.83±0.02^b,c^	1.82±0.07^a^	nd	0.28±0.01^a,b^	3.27 ±0.05^a,e,f^	nd	nd	nd	8.46	5.37

*Control: spontaneous fermentation.

**MIX: *L. brevis* ZF165; *W. cibaria* KKP2058; *L. plantarum* KKP1822; *L. fermentum* KKP1820; *Leuconostoc mesenteroides* JEIIF.

***BAI: biogenic amine index calculated acc. to Świder et al.^16^, BAI = putrescine + cadaverine + histamine + tyramine.

Different letters indicate significant differences among the mean values according to the least significant difference test (*P* ≤ 0.05).

Among them, putrescine was at the highest level (4.59 and 3.82 mg·L^−1^ for Wodan and Alto, respectively). Putrescine is an aliphatic polyamine derived from enzymatic decarboxylation of ornithine or arginine mediated via ornithine decarboxylase (DOC). Following the polyamines synthesis pathway, putrescine can be further converted by spermidine synthase into spermidine by the addition of an aminopropyl group. An addition of another aminopropyl group to the spermidine results in the formation of spermine^29^. These three polyamines are important components of living organisms because they are involved in a variety of biochemical processes related to cellular metabolism including apoptosis, cell division, and differentiation, cell proliferation, DNA and protein synthesis, gene expression, homeostasis, and signal transduction^30^. However, when they are in excessive amounts can have a toxic effect but less than histamine and tyramine, which are recognized by EFSA to be the most harmful BA^31,32^. The negative activity of polyamines is mainly related to the potentiation of the toxicity of other amines, especially histamine. Considering the concentration of polyamines in the studied fresh juices, Wodan was characterized by a higher level of putrescine than Alto, which, on the other hand, had higher amounts of spermidine and spermine. In the case of spermine and spermidine, Wodan had more spermine than spermidine (0.27 mg·L^−1^ vs. 0.15 mg·L^−1^) while, the opposite was found in Alto (0.32 mg·L^−1^ vs. 0.46 mg·L^−1^ for spermine and spermidine, respectively). No histamine and cadaverine were found in fresh red beet juices. Tyramine level was comparable between both cultivars and was 0.63 mg·L^−1^ for Wodan and 0.61 mg·L^−1^ for Alto. The fermentation process substantially affected the BA profile of fresh red beet juices. The concentrations of individual BA found in fresh juices changed depending on the used culture starter. Additionally, two other BA *i.e.* histamine and cadaverine were detected. Histamine was found in the spontaneously fermented juices and inoculated with *L. brevis* ZF165 Alto and Wodan, and Wodan inoculated with *W. cibaria* KKP2058 at levels below 1 mg·L^−1^. In the other cases *i.e.* samples of both cultivars inoculated with MIX, and Alto fermented with *W. cibaria* KKP 2058 no histamine was detected. The highest amount of histamine was found in spontaneously fermented Wodan juices, 0.52 mg·L^−1^, and its content reduced about twofold when single starters were used. The juices from both fermented cultivars were characterized by considerably higher levels of tyramine than histamine and tyramine dominated the other BA found in the fermented red beet juices. The highest concentration of tyramine was obtained for samples obtained with *L. brevis* ZF165, regardless of the cultivar used (12.54 and 16.06 mg·L^−1^ for Wodan and Alto, respectively). Simultaneously, these samples had the lowest amount of tyrosine, a tyramine precursor (Table 3). Thus, these results indicate that the microbial community of the samples inoculated with *L. brevis* ZF165 showed the highest activity in tyrosine decarboxylation. The microbial community of samples treated with *W. cibaria* KKP2058 and MIX produced significantly lower amounts of tyramine, especially in the case of the Alto cultivar. The second dominating BA in the juices of fermented red beet was putrescine followed by spermidine. It is interesting to note that in the case of putrescine, different effects were observed depending on the cultivar. The fermented Wodan samples had a lower putrescine content than the fresh juices, while that of Alto were on comparable levels or higher. Moreover, the fermented Alto juices characterized a higher level of this BA than that of the Wodan cultivar, regardless of the strain used. The highest amount of putrescine in both cultivars was determined for juices treated with *L. brevis* ZF165, similar to tyramine. Whereas, its lowest content showed samples fermented with MIX. These samples had instead the highest content of spermidine. The content of spermidine in the other fermented juices of both cultivars was on a comparable level. The spermine content in all cases was about ten times lower than that of its precursor—spermidine, indicating low activity of spermine synthase.

In the case of cadaverine, its highest amounts were detected in the juices from spontaneously fermented and inoculated with *L. brevis* ZF165 Wodan red beets. As in the previous case, these samples were characterized by the lowest amounts of the cadaverine precursor, *i.e*. lysine (Table 3).

The calculated biogenic amines index (BAI) used as a quality indicator of fermented beetroots, acc. to Świder et al.^16^, was the highest for juices obtained during fermentation with *L. brevis* ZF165 *i.e.* 17.05 and 22.29 mg·L^−1^ for Wodan and Alto, respectively (Table 4). The relatively high value of the BAI for these samples resulted mainly from the high level of tyramine.

The presented results are in the line with the previous observation that the formation of BA is complex and dependent on various factors including raw material composition (red beet variety), and changes in the microbial composition due to the interspecific competition during fermentation. The obtained metagenomic data revealed the presence in the microbial profile of the fermented red beet juices both LAB as well as Gram-negative bacillus belonging to the order *Enterobacterales i.e. Serratia, Kosakonia, Enterobacter*, which are also known BA producers. Bunkova et al.^33^ reported that *Enterobacteria* were found to be the largest producers of amines among the studied Gram-negative bacteria in cultivation broth. In another study performed by Li et al.^34^, a positive correlation between the *Serratia* level and the formation of spermine, and cadaverine during the fermentation of the soybean paste has been observed. This finding has been related to the high lysine decarboxylase and ornithine decarboxylase activities of this group of microorganisms.

### Total phenolic content

The total phenolic content of fresh juices of Wodan and Alto cultivars was 1274.6 mg GAE·L^−1^ and 1177.3 mg GAE ·L^−1^, respectively (Table 5). The total phenolic content of red beet juices of Wodan cultivar reported by Czyżowska et al.^10^ was about 800 mg·L^−1^. The differences in the obtained concentrations can be related to the different methods and regions of cultivation. It was already suggested by the other authors that weather conditions and agrotechnical treatments strongly affect the content of phenolic compounds or other bioactive compounds, and thus the observed differences in their content between the same and different varieties of red beet^35,36^. The total phenolic contents of juices obtained during fermentation were significantly lower than that of fresh juices (Table 5). A higher decrease was observed for the spontaneously fermented samples of Alto. In contrast, the total phenolic content of juices from spontaneously fermented Wodan red beets was distinctly higher even than that of inoculated with *W. cibaria* KKP2058 and MIX. It should be noted that the used Folin Ciocalteu phenol reagent is reactive not only towards phenols but also to other classes of compounds^37^. Recently, it was hypothesized that it can be also specific to betalain^35^. In our study, the level of betalains in fermented Wodan beetroot juices is higher than that of Alto, especially in spontaneously fermented beetroot. Moreover, in most cases, *i.e.* in all fermented Alto samples and inoculated with *W. cibaria* KKP2058 and MIX Wodan samples, a higher betalain content corresponds to a higher total phenolic content. However, the different results obtained for juices of spontaneously fermented beetroot and that inoculated with *L. brevis ZF165* suggest that the findings should be considered taking into account the complexity of the studied matrix and the specificity of the Folin Ciocalteu reagent.

**Table 5:** The total phenolic content and antioxidant activity of juices obtained during spontaneously and inoculated fermented red beet juice.

Sample	Total phenolic content [mg GAE·L^-1^]	DPPH [µmol Trolox·mL^-1^]
Wodan (fresh)	1274.6 ± 15.3^c^	0.99 ± 0.01^d^
Wodan (control)*	690.3 ± 30.1^b^	0.54 ± 0.05^b^
Wodan (*L. brevis* ZF165)	766.0 ± 68.3^b^	0.48 ± 0.04^a^
Wodan (*W. cibaria* KKP2058)	581.7 ± 36.9^a^	0.56 ± 0.02^b^
Wodan (MIX)**	585.4 ± 33.2^a^	0.67 ± 0.05^c^
Alto (fresh)	1177.3 ± 17.9^c^	0.93 ± 0.01^d^
Alto (control)*	524.1 ± 52.2^a^	0.26 ± 0.07^a^
Alto (*L. brevis* ZF165)	534.9 ± 12.3^a^	0.38 ± 0.01^b^
Alto (*W. cibaria* KKP2058)	564.5 ± 66.6^b^	0.49 ± 0.03^c^
Alto (MIX)	573.6 ± 17.2^b^	0.55 ± 0.05^c^

*Control: spontaneous fermentation.

**MIX: *L. brevis* ZF165; *W. cibaria* KKP2058; *L. plantarum* KKP1822; *L. fermentum* KKP1820; *Leuconostoc mesenteroide*s JEIIF.

### Antioxidant activity

The antioxidant activity (AA) of the studied fresh juices, 0.99 µmol Trolox·mL^-1^ for Wodan and 0.93 µmol Trolox·mL^−1^ for Alto decreased significantly after 7 days of fermentation (Table 5).

The fermented Wodan samples were characterized by a higher value of this parameter than Alto. The highest value of AA was detected in juices of both cultivars obtained during fermentation with MIX, while the lowest was those of spontaneously fermented Alto. Czapski et al.^38^ observed a high correlation between the AA and red colorants content in the studied raw juices of red beets and concluded that the AA is mainly determined by betacyanins. Similarly, Sawicki, & Wiczkowski^11^ reported a decrease in betacyanins concentration and AA during the spontaneous fermentation of beetroots. The AA of betacyanins has been related to the presence of free phenolic hydroxyl groups in their structure. In our study, the AA varied depending on the used starter culture indicating the diverse metabolic activity of the LAB strains towards bioactive compounds acting as free radical scavengers. The observed reduction of betacyanins and AA is in line with the observation made by the other authors.

## Conclusion

The results obtained in the present study showed different metabolic activities in relation to the starter cultures toward the analyzed bioactive compounds. The observed differences also depended on the used red beet cultivar. Generally, in most cases, the use of starter culture led to a significant reduction of betalains and free amino acid content. However, in the case of betalains the use of a multi-culture starter allowed to largely prevent their reduction. Inoculation of red beets reduced the relative abundance of the pathogens in the case of single-strain cultures, and their limitation in the case of multi-strain cultures. No histamine formation was observed in juices obtained after the fermentation of red beets with the multi-culture starter. In other cases, the fermented juices were characterized by a higher level of biogenic amines than fresh red beet juices. The antioxidant activity of obtained during fermentation juices was lower than that of fresh juices and to some extent correlated with the total phenol content and betacyanin level. Thus, the use of starter cultures allows to control the fermentation process by limiting the growth of undesirable organisms, however, on the other hand, it leads to changes in the content of beneficial compounds and antioxidant activity of the fermented product. Considering the above results, a balance should always be kept between the safety, quality, and acceptability of the final product when designing an appropriate starter culture.

## Materials and methods

### Chemicals/Reagents

The LC–MS grade acetonitrile, LC–MS water, and hexane were purchased from Witko (Łódź, Poland). Di-sodium tetraborate (borax) ≥ 99% was supplied by Chempur (Piekary Śląskie, Poland). Ammonium formate ≥ 97% and formic acid assay 98–100% were purchased from Chem-Lab (Zedelgem, Belgium). Dansyl chloride 97% was purchased from abcr GmbH (Karlsruhe, Germany). Pure trichloroacetic acid was supplied by POCH (Gliwice, Poland). Certified analytical standard (agmatine ≥ 97%, putrescine, histamine, cadaverine, tryptamine, phenylethylamine, tyramine, spermidine, spermine, arginine, ornithine, glutamine ≥ 99%, histidine, lysine, tryptophan ≥ 98%, phenethylamine, tyrosine, 1,7-diamino heptane assay 98%, and ammonium hydroxide solution ~ 25%) were supplied by Sigma-Aldrich (Darmstadt, Germany). The Folin Ciocalteau reagent, DPPH, Trolox, and gallic acid, methanol, and sodium carbonate were purchased from Sigma-Aldrich.

### Plant materials

Fresh red beetroot (*Beta vulgaris* l. subsp. *vulgaris*) of the cultivar Alto and Wodan were purchased from a local vegetable market in Poland. The experiments were generally performed after procurement.

### Microorganisms

The used strains of lactic acid bacteria i.e. *W. cibaria* KKP2058, *L. brevis* ZF165, *L. plantarum* KKP1822, *L. fermentum* KKP1820, *Leuconostoc mesenteroides* JEIIF isolated from various plant sources were obtained from the Culture Collection of Industrial Microorganisms, prof. W. Dąbrowski Institute of Agricultural and Food Biotechnology-State Research Institute in Warsaw, Poland.

### Fermentation

Before the fermentation, whole red beetroots were washed, peeled, and cut into slices 2–3 mm thick. Then, the slices were placed into sealed glass jars, flooded with marinade (100 mL) containing salt (1.5%), and inoculated with a fresh culture containing 1 × 10^5^ CFU mL^-1^. The control sample was prepared by spontaneous fermentation without the addition of starter cultures. During the fermentation jars with red beetroots were kept at room temperature for 7 days. After 7 days, the fermentation process ended, and the juices obtained from fermented red beetroots were collected and frozen. The LAB count (counted on MRS agar) at the end of fermentation was about 7–8 log CFU·mL^−1^, regardless of the fermentation conditions (with/without starter culture).

### DNA extraction and 16S rDNA amplicon sequencing (NGS)

Total DNA was isolated using QIAamp PowerFecal Pro DNA Kit (Qiagen) in line with the manufacturer's instructions. DNA purity was measured by the Nanodrop ND-1000 Spectrophotometer (ThermoFisher Scientific), and DNA concentration was quantified by a Qubit 4.0 Fluorometer using the Qubit dsDNA BR Assay Kit (Invitrogen). Library preparation, dilution, and sequencing were prepared according to the method described by Juszczuk-Kubiak et al.^39^. PCR amplification of 16S rDNA genes for each sample, 5 ng of total DNA, and one pair of primers (341F: CCTACGGGNGGCWGCAG-3′; 805R: GACTACHVGGGTATCTAATCC-3′) was used to amplify the V3-V4 region of the 16S rDNA gene. ReadyMix (KAPA Biosystems) and 10 μM of each forward and reverse primer. PCR assay consisted of 95 °C/3 min, 25 cycles of 95 °C/30 s, 56 °C/30 s, and 72 °C/30 s, followed by 72 °C/5 min. A negative control without template DNA was also included in the PCR assay. Amplicons were purified using MagSi-NGSPREPPLUS (Steinbrenner Laborsysteme) and quantified using the Qubit DNA BR Assay Kit (Invitrogen) in conjunction with a Qubit 4.0. Library preparation and sequencing of the 16S rDNA fragmenwerewas indexed using the Nextera XT kit (Illumina) according to the Nextera DNA Sample Preparation Guide (protocol #15,044,223). Each index PCR reaction contained 5 μL of the i7 and i5 adapter, 10 μL of KAPA HiFi HotStart ReadyMix, 20 μL of template DNA (7.0 ng/μL), and 10 μL of H2O for a total reaction volume of 50 μL. The indexed PCR was cycled according to the Nextera DNA Sample Prep Guide, and the libraries were cleaned up using MagSi-NGSPREPPLUS (Steinbrenner Laborsysteme). The DNA libraries were quantified using the Qubit 4.0 along with the Qubit DNA HS Assay Kit, and the quality was assessed on a TapeStation 4200 using the High Sensitivity D1000 SreenTape Assay Kit (Agilent). Indexed libraries were normalized to 4 nM and pooled. The normalized, pooled 4 nM library was denatured using 0.2 N NaOH and diluted to 10 pM using prechilled HT1 buffer supplied in the Nextera XT Kit (Illumina). The 10% of denatured PhiX library (Illumina) was spiked into the denatured and indexed library, which was loaded into Illumina Miseq v3 reagent cartridge, and 16S rDNA gene amplicons was sequenced on an Illumina MiSeq platform using the 600 cycles (2 × 300 bp) v3 chemistry.

### Sequencing data analysis

Bioinformatic analysis was performed using the CLC genomic workbench v.8.5.1 with Microbial Genomics Module (Qiagen)^40^. Briefly, for microbial community analysis, obtained raw paired-end reads were assembled and filtered for quality (max error rate 1%) and length (minimum 300 bp of merged reads). Low-quality scores and chimeric reads were deleted using the USEARCH (http://www.drive5.com/usearch ). The merged sequences were trimmed of barcodes and primers and next the high-quality sequences were clustered into operational taxonomic units (OTUs). A sensitive BLASTN search against the GreenGenes 16S sequence database, v.13.5 (http://greengenes.lbl.gov to obtain taxonomy assignment was utilized.

### Determination of betalains with HPLC analyses

#### Quantitative analysis of betalains

Fermented juices were analyzed directly after filtration through a 0.45 μm syringe filter. The analyses were conducted acc. to the method reported by Ravichandran et al.^41^ using Sunfire C8, 5 μm, 4.6 × 250 mm column (Waters) with an appropriate pre-column. The separation of 10 μL samples was performed within 60 min at a column temperature of 30 °C and a flow rate of 1.0 mL·min^-1^ using a gradient of 0.2% formic acid (A) and acetonitrile (B) as follows: A:B[%]; 100:0 (0 min.); 100:0 (0–7 min.); 97:3 (7–17 min.); 90:10 (17–27 min.); 90:10 (27–35 min.); 80:20 (35–45 min.); 0:100 (45–50 min); 100:0 (50–55 min.); 100:0 (55–60 min.). The pigments were quantified using spectrophotometric data: for betacyanins at a wavelength of 538 nm (expressed as betanin equivalents), while for betaxanthins at a wavelength of 480 nm (expressed as vulgaxanthin I equivalents).

#### Identification of betalains

The abovementioned method of separation was repeated for selected samples using other modes of detection: fluorescence (at an excitation wavelength of 465 nm and emission wavelength of 510 nm) and mass spectrometry (at *m/z* range of 250–800). The most abundant pigments were identified based the on results of our previous experiments^42^ and literature data on *m/z* ratios and typical retention order during RP-HPLC separation^43–45^. Additionally, betacyanins and betaxanthins were distinguished based on their fluorescence at tested wavelengths. In betaxanthins, strong fluorescence occurs due to the presence of four conjugated double bonds, while coupling them to the aromatic ring of cyclo-DOPA in betacyanins leads to a lack of fluorescence^46^.

### Determination of free amino acids and biogenic amines content with UPLC analyses

Biogenic amines (histamine, tyramine, putrescine, cadaverine, tryptamine, spermine, spermidine, agmatine, and 2-phenethylamine) and free amino acids (histidine, lysine, phenylalanine, tyrosine, arginine, ornithine, glutamine, isoleucine, leucine, valine, threonine, asparagine, serine, methionine, proline, aspartic acid, glutamic acid) were analyzed according to the methods described by Świder et al.^16^ with some modification.

#### Sample preparations for UPLC analysis

An aliquot (2 g) of the juices obtained from fermented red beetroots was weighed into a 50 mL centrifuge tube, spiked with 50 μL of 1,7-diaminoheptane internal standard solution (1 mg·mL^−1^) and with 40 mL of 5% trichloroacetic acid, and then shaken and centrifuged at 10,000 × *g* for 10 min. The supernatant was filtered through a filter paper. One milliliter of distilled water, 1 mL of borax solution (5%), and 100 μL of the sample supernatant were mixed together in a 15 mL polypropylene tube. Dansyl chloride (2.5 mL, 20 mM) dissolved in acetonitrile was added, and the mixture was shaken up and put in a shaking water bath operated at 30 °C for 1 h in the dark. Then, 125 μL of ammonia solution (400 mM) was added, and the tube was left intact for 15 min in a dark place. Finally, the mixture was filtered through a 0.45 μm syringe filter into a chromatographic vial for analysis with UPLC.

#### UHPLC-MS/MS (Orbitrap) analysis

Acquity H-Class ultra-high-performance liquid chromatography coupled to a high-resolution mass spectrometer Q Exactive Orbitrap Focus MS (Thermo Fisher Scientific, U.S.) was used for the analysis. The separation of free amino acids and biogenic amines was performed on a nonporous 100 × 2.1 mm Cortecs UPLC C18 1.6 µm column (Waters, Milford, U.S.) with a flow rate of 0.3 mL·min^-1^ after injecting aliquots (5 μL) of sample solution. The elution was conducted using a gradient system containing solvent A (water/acetonitrile: 90/10) and solvent B (acetonitrile/water: 90/10), each with 5 mM of ammonium formate and 1% formic acid. Gradient A:B (%) was as follows: 90:10 (0–2 min.)—waste, 0:100 (2–22 min.), 0:100 (22–25 min.), 90:10 (25–26 min.), and 90:10 (26–28 min.). The analysis was based on scanning in the positive ionization mode, with the h identification of amino acids and biogenic amines being optimal under the following conditions: heated electrospray ionization (HESI): 3 kV, capillary temperature: 256 °C, sheath gas flow rate: 48, auxiliary gas flow rate: 11, sweep gas flow rate: 2, probe heater temperature: 413 °C. Scan range: 200–1200 m/z (Full MS), 80–1000 m/z (AIF). Linearity (as evaluated with standard solution) was in the 10^3^ range, typical instant resentment of that type.

### Xcalibure 4.2.47 software was used to acquire and analyze data

The established during validation limits of quantification (LOQ) of the studied BA expressed in mg·L^-1^, are as follows: tryptamine (0.29); putrescine (0.03); spermine (0.08); cadaverine (0.06); histamine (0.09); tyramine (0.07); spermidine (0.08); agmatine (0.14); 2-phenethylamine (0.35).

### Determination of total phenolic content

The total phenolic content was determined using the Folin Ciocalteu approach^47^. Briefly, 1 mL of the diluted in water (1:4) fermented beetroot juice was added to 5 mL of Folin Ciocalteu reagent. After standing at room temperature for 3 min., 4 mL of sodium carbonate (% *w/v*) was added to the mixture. Then the samples were allowed to stand at room temperature for 60 min., and then the absorbance was measured at 765 nm on a DU 800 UV/VIS spectrophotometer (Beckman Coulter, Inc.). Total phenolic content was expressed as gallic acid equivalents (GAE) (mg GAE·L^-1^) in the linear range of the standard of 100–500 mg·L^-1^ (R^2^ = 0.998).

### Determination of antioxidant activity by DPPH methods

The DPPH assay was performed acc. to Guldiken et al.^48^. Briefly, 100 μL of diluted in water (1:4) sample of fermented beetroot juices was mixed with 3.9 mL of fresh prepared methanolic solution of DPPH (1 × 10^–3^ M), and then incubated in the darkness at room temperature. After 30 min, the absorbance was measured at 517 nm using DU 800 UV/VIS spectrophotometer (Beckman Coulter, Inc.). The standard curve was plotted based on Trolox concentrations within the range of 0.01–1.5 mM. The antioxidant capacity was expressed as µM Trolox·mL^−1^.

### Statistical analysis

The results were analyzed using Statistica ver. 8.0 software (Statsoft, USA). The compatibility of variable distribution with normal distribution was verified with the Shapiro–Wilk test, while the hypothesis about the homogeneity of variance was tested with the Levene test and Brown-Forsythe test. To compare the mean values of particular parameters, an ANOVA was performed and a post hoc analysis (Tukey test). The level of significance was 0.05.

## Supplementary Information

Supplementary Information 1.

Supplementary Information 2.

## Data Availability

The data that support the findings of this study are available from the corresponding author upon reasonable request. The datasets analyzed during the current study are available in the GenBank repository, KKP 2058—MN736626 (https://www.ncbi.nlm.nih.gov/nuccore/MN736626); KKP 1822—ON929305 (https://www.ncbi.nlm.nih.gov/nuccore/ON929305); ZF 165—ON929884 (https://www.ncbi.nlm.nih.gov/nuccore/ON929884); JEIIF—ON929306 (https://www.ncbi.nlm.nih.gov/nuccore/ON929306).
