# Supplementary material for: Starter culture-related changes in free amino acids, biogenic amines profile, and antioxidant properties of fermented red beetroot grown in Poland
Source: Sci Rep. 2022 Nov 21;12:20063. doi: 10.1038/s41598-022-24690-9 (PMC9681880; doi:10.1038/s41598-022-24690-9)
Supplement: Supplementary file 1 — Supplementary Information 1. [file 41598_2022_24690_MOESM1_ESM.doc]

Table S1. Data of found for betalains in fresh and fermented red beet juices of Wodan and Alto variety.

| Compound | λ_max_  [nm] | Retention time  [min.] |
| --- | --- | --- |
| Vulgaxanthin I | 468 | 15.3 |
| Vulgaxanthin II | 470 | 19.5 |
| 2-O'-glucosyl-betanin | 531 | 25.8 |
| Betanin | 534 | 26.2 |
| Isobetanin | 534 | 29.2 |
| Betanidin | 543 | 33.1 |
| Isobetanidin | 543 | 39.0 |
| neobetanin | 463 | 42.8 |
