# Supplementary material for: Starter culture-related changes in free amino acids, biogenic amines profile, and antioxidant properties of fermented red beetroot grown in Poland
Source: Sci Rep. 2022 Nov 21;12:20063. doi: 10.1038/s41598-022-24690-9 (PMC9681880; doi:10.1038/s41598-022-24690-9)
Supplement: Supplementary file 2 — Supplementary Information 2. [file 41598_2022_24690_MOESM2_ESM.docx]

Table S2. Comparison of betalains changes in the studied fresh and fermented red beet juices of Wodan and Alto variety expressed by betanin+isobetanin/betanidin+isobetanidin, B+I/Bd+Id (ß-glucosidase index), isobetanin/betanin, I/B (isomerization index), neobetanin/betanin, N/B (dehydrogenation index), and vulgaxanthin I/betanin, V/B ratio, acc. to Czyżowska et al. [26]. Control denotes spontaneous fermentation.

| Sample | (B+I/Bd+Id) | I/B | N/B | V/B |
| --- | --- | --- | --- | --- |
| Wodan (fresh) | 0.00 | 0.10 | 0.07 | 0.15 |
| Wodan control | 0.23 | 0.05 | 0.04 | 0.38 |
| Wodan (*L. brevis* ZF165) | 0.24 | 0.20 | 0.08 | 0.39 |
| Wodan (W. cibaria KKP2058) | 2.05 | 0.11 | 0.04 | 0.08 |
| Wodan (MIX) | 17.07 | 0.13 | 0.03 | 0.03 |
| Alto (fresh) | 0.00 | 0.12 | 0.08 | 0.18 |
| Alto control | 0.37 | 0.09 | 0.00 | 0.14 |
| Alto (*L. brevis* ZF165) | 0.21 | 0.13 | 0.01 | 0.36 |
| Alto (W. cibaria KKP2058) | 1.42 | 0.12 | 0.01 | 0.06 |
| Alto (MIX) | 26.02 | 0.15 | 0.00 | 0.03 |
